# Plant Functional Traits or Microbiomes Associated with Diseases, Pests, Human Activities and Climate Change

**DOI:** 10.3390/plants15020238

**Published:** 2026-01-13

**Authors:** Jian-Wei Guo, Honglan Yang, Xiaolin Wang

**Affiliations:** 1 College of Agronomy and Life Sciences, Yunnan Key Laboratory of Konjac Biology, Yunnan Urban Agricultural Engineering and Technological Research Center, Kunming University, Kunming 650214, China; gjwkf475001@sina.com; 2 State Key Laboratory of Ecological Safety and Sustainable Development in Arid Lands, Xinjiang Institute of Ecology and Geography, Chinese Academy of Sciences, Urumqi 830011, China; 3 China-Tajikistan Belt and Road Joint Laboratory on Biodiversity Conservation and Sustainable Use, Urumqi 830011, China; 4 College of Agriculture, South China Agricultural University, Guangzhou 510642, China

## 1. Introduction

The ongoing global climate change is resulting in increases in CO_2_, temperature, humidity, salinity, flooding, and drought, driving subsequent rises in the prevalence, dispersal, and range of different plant pathogens. Thus, understanding how plants adapt to abiotic or biotic stress through trait changes has become imperative [1,2,3,4]. Drought and graze stress can contribute to an increase in trait diversity, which challenges the traditional view that harsh environmental conditions reduce plant trait diversity [5]. Additionally, the national average rate of crop pest and disease (CPD) increased by four times in China between 1970 and 2016; moreover, warmer nighttime temperatures are a key driver of the increasing occurrence of CPDs [6]. A plant’s functional traits, including its morphological, physiological, and biochemical characteristics, are crucial in determining its response to its environment [7]. Beyond inherent trait adaptations, plants can recruit specific microbial taxa to adapt to harsh environments. In return, the microbiome can improve the fitness of host plants under harsh environments by activating genes involved in nutrient acquisition and stress tolerance [8].

The plant microbiome, encompassing rhizosphere, epiphytic, and endophytic microbiomes, has demonstrated great potential in agricultural systems, in which it may offer desirable agronomic and ecological functions [8]. For instance, germinating seeds can secrete benzaldehyde into soil, resulting in enrichment of *Enterobacteria* bacteria. *Enterobacteria* and *Weeksellaceae* notably improve leaf length, root length, and biomass compared with seeds which lack these microbes [9], and endophytic seed bacteria *Sphingomonas melonis*, accumulated and transmitted across generations, can confer host resistance to the seed-borne pathogen *Burkholderia plantarii* by producing anthranilic acid [10]. On the contrary, the AGR-encoded lineage-specific effector *Ave1* in the soil-borne fungus *Verticillium dahliae* can manipulate the micriobiome and inhibit *Sphingomonadales* antagonistic effects on *V. dahliae* to promote fungal virulence in plants [11]. Meanwhile, Av2, which exerts selective antibacterial activity on the growth of antagonistic *Pseudomonas* spp., suppresses the recruitment of *P. aeruginosa* by inhibiting the plant’s cry-for-help response, and this was found to weaken infected tomato plants’ defenses through the microbiome [12]. During the past few decades, the importance of plants’s functional traits and how the microbiome responds to diseases, pests, and climate change has been overstated. More attention has been paid to the utilization of beneficial microbial communities or plants with specialized functional traits as sustainable strategies for disease and pest management, as well as climate change adaptation [13]. The application of signal molecules (e.g., flavonoids, monocotyledons, coumarins, glucosinolates, benzoxazolones, jasmonic acid) and synthetic microbial communities can effectively improve nitrogen fixation efficiency and drought resistance in plants [14,15,16]. Moreover, when infected with SRBSDV (Southern rice black-streaked dwarf virus), rice is not a passive victim but actively reshapes its rhizosphere ecosystem by changing secondary metabolites to form a new steady state, attracting beneficial bacteria through flavonoids (such as quercetin) and simultaneously expelling potential pathogens or disruptors [17]. Similarly, the repellent plant *Rosmarinus officinalis* and its volatile compounds significantly reduced infestations and egg-laying by the tomato leaf miner (*Tuta absoluta*) in greenhouses, decreasing tomato damage rates by 40–50% [18]. However, research exploring the associations between plant functional traits and microbiomes and plant diseases, pests or climate change, particularly with regard to their roles in plant vulnerability and stress adaptation processes, remains relatively scarce.

This topic, “Plant Functional Traits or Microbiome Associated with Diseases, Pests, and Climate Change”, provides a comprehensive platform for showcasing the latest research and insights into plants’ adaptive strategies amidst rising temperatures, aridification, and biotic threats. We aim to facilitate interdisciplinary collaboration at the frontiers of plant biology, ecology, and microbiology, generating innovative solutions that safeguard the sustainability of global food systems and ecosystems. Relevant research topics include multiple-pathogen identification of plant diseases, the development and application of disease-resistant genes, the dual effect of selenium on improving disease resistance and fruit quality, the screening and evaluation of drought resistance in cotton germplasm resources, as well as the influence of plant functional traits on the geographic patterns of herbivory.

## 2. Multiple-Pathogen Identification of Plant Diseases

Guo et al. reported that ring rot, brown spot, black spot, and soft rot of loquat fruit (*Erilbotrya japonica*) were caused by *Botryosphaeria dothidea*, *Trichothecium roseum*, *Alternaria alternata*, and *Pestalotiopsis kenyana* in Yunnan, China, respectively. Moreover, all of the tested strains exhibited higher virulence on wounded fruits than non-wounded ones, and virulence varied with species (and even among strains), temperature, and humidity [19]. Albuquerque et al. discovered that more than 19 fungal species may be responsible for almond decline and dieback via metabarcoding and traditional culture plate isolation, likely originating from nursery substrates or soils shared with other crops; thus, it has been deemed urgent to test the pathogenicity following Koch’s postulation [20]. These findings highlight the urgent need to determine the dominant pathogen and characterize pathogen diversity in horticultural plants across different regions, which has been studied in far less depth than agricultural crops, across different regions. This should be achieved using a traditional pure cultivation method combined with high-throughput sequencing (HTS)-based whole-genomic and metagenomic analysis [8,21], which would achieve a perfect combination of correlation analysis indication and causal relationship verification and provide a theoretical basis for the development of colloidal gold immunoprecipitation technology, epidemic, more widespread development and application of disease-resistant germplasm resources and their genes, and the control of plant diseases [22].

## 3. Screening and Application of Stress-Resistant Genes, Germplasm Resources, or Trace Elements

The seven sets of markers for the *Pik* locus, a well-studied disease resistance (R) gene locus in rice that provides resistance to specific strains of the blast fungus *Magnaporthe oryzae*, associated with rice blast resistance, were explored in an analysis of 163 japonica rice cultivars bred in Yunnan Province between 1980 and 2020. The analysis revealed that 63 cultivars contained *Piks* (accounting up 38.65%), 61 cultivars carried the novel *Pik* locus haplotype (37.42%), and 35 cultivars did not contain any of the seven *Pik* locus alleles/haplotypes (21.47%); 1.84% contained *Pikm*, 0.61% contained *Pik*, and 0% contained *Pi1*, *Pikp*, and *Pikh*, respectively [23]. Wang et al. conducted an objective and comprehensive evaluation of 502 cotton accessions for drought tolerance during the germination stage to quickly establish a set of accurate, comprehensive, and rapid drought tolerance identification systems for cotton; furthermore, HS120 was identified as a strong contributor to drought accession [24]. These results provide the germplasm resource foundation for the comprehensive utilization of resistance genes via gene-editing technology, because the effect of a single gene on improving crop yields—including enhancing drought or disease resistance—is limited [25]. Additionally, Liu et al. found that the applications of selenium (100 mg/plant) in the field not only significantly reduced the disease index of banana wilt, but also increased the total selenium content in banana pulps 23.7-fold [26]. Together, these advances underscore the importance of integrated strategies—combining genetic resources, germplasm screening, and agronomic practices—for sustainably enhancing crop resilience under biotic and abiotic stresses.

## 4. Plant Functional Traits Response to the Environment

Ji et al. demonstrated that plant functional traits, climatic factors, and soil nutrients explained 7.3%, 4.66%, and 0.98% of the latitudinal variation in herbivory, respectively. Correspondingly, herbivory decreased with increasing latitude from the equator to the poles, supporting the latitudinal herbivory hypothesis that the intensity of herbivory (i.e., plants’s consumption by herbivores) should decrease as latitude increases [27]. Another study discovered that within-population variation of herbivores increases with latitude and decreases with plant size, and it exhibits a phylogenetic structure, according to herbivorous surveys of 503 plant species across 790 locations across 116 latitudes [28]. Thus, the effect of latitude on plant functional traits and the population variation of herbivores is still in need of further exploration.

## 5. Outlook

Dozens to hundreds of fungal species from diseased plant tissues have been identified by microbial taxonomists based on morphological and multi-locus phylogeny [29,30]. In a study focused on sugarbeet in the United States, meta-transcriptomic analysis revealed the presence of four known viruses and one new one [31]. The quantitative microbiome revealed a close correlation between infection with soybean green syndrome and a novel recombinant twin virus, which is now referred to as soybean green syndrome-related virus [32]; these findings were confirmed by Koch’s postulations [33], and the virus was detected in 368 green syndrome samples collected from 17 regions across eight provinces in China [34]. The integration of quantitative microbiome data with Koch’s postulates enables more accurate and timely identification of phytopathogens, as well as improved monitoring and tracking of their hosts, geographic distribution dynamic, epidemiology, and population structure. On this basis, the development and application of rapid diagnostic technologies and disease-resistant varieties tailored to dominant phytopathogens in specific regions will attract increasing amounts of attention.

At the community level, species with a “fast strategy” (resource-acquisitive types), characterized by rapid growth and high mortality, are replenished quickly, whereas “slow strategy” species (resource-conservative types) with slow growth and low mortality are replenished slowly. This growth–mortality trade-off is widely regarded as an important mechanism for species’ coexistence. However, a recent study showed that the classic ecological trade-off rules may not fully apply in subtropical monsoon forests that are frequently affected by typhoons. Instead, the tress growth, mortality, and recruitment processes of trees are relatively independent and driven by different combinations of functional traits. Among these, hydraulic structure governs growth, leaf defense traits determine survival, and the overall “fast resource acquisition” strategy dominates forest regeneration [35]. Furthermore, while the relationships between plant communities and spatial scale characteristics and the environment have been widely explored [36], the temporal stability of these trait–environment correlations remains critical for future ecological predictions. Famiglietti et al. found that leaf traits are mainly influenced by recent climate fluctuations, while wood density more accurately predicts historical climate conditions [37]. In addition, both plant and fungal diversity are key to maintaining ecosystem multifunctionality, and in low-diversity plant communities, ecosystem multifunctionality is particularly sensitive to changes in fungal diversity, as these communities lack the ability to compensate through strong selection effects. Diversified fungal communities can help alleviate the negative impact that plant diversity loss has on ecosystem multifunctionality, and plant diversity can, in turn, alleviate the negative effects of fungal diversity loss by promoting the systematic development, aggregation, and functional redundancy of fungal communities [38]. Therefore, the key functional traits for plant growth and survival and the influence of plant microbiome on the key functional traits on the spatiotemporal scale are worthy of attention.

At the species level, diversity is the strongest factor driving trait variation, while environmental stress mainly amplifies intraspecific rather than interspecific variation [39]. Additionally, from temperate to subtropical regions, the intraspecific trait variation of root traits, especially root diameter and specific root length, decreases as the latitude lowers, whereas it increases in response to increases in temperature and precipitation. The closer one is to the equator, the greater the individual differences in plant roots. This latitudinal gradient is consistent with the global distribution pattern of biodiversity: a high-latitude (cold and dry) environment with strong environmental filtering results in individual plant convergence and low variation, whereas a low-latitude (warm and humid) environment with intense competition and resource heterogeneity promotes the coexistence of plants through diversified traits [40]. Similarity, based on an analysis of pathogen load, climatic factors, and plant biomass across a vast grassland ecosystem spanning 4000 km, Dang et al. found that temperature is the main factor driving changes in disease load. Shrubs’s expansion reduces disease prevalence in cold regions, while it may increase the presence of disease in warm regions [41]. Otherwise, the composition of plant rhizosphere microbial communities exhibits distinct local nitrogen adaptation characteristics: plant genotypes from low-nitrogen environments tend to recruit nitrogen-fixing rich microbial communities, which in turn enhance plant growth adaptability under nitrogen-deficient conditions. In nitrogen-rich environments, the relative abundance of nitrifying bacteria and ammonia oxidizing microorganisms in the plant rhizosphere is higher. These microorganisms can oxidize ammonium nitrogen to nitrate nitrogen, which accelerates nitrogen cycling but may also cause nitrogen loss. This suggests that the interaction between plants and microorganisms has a significant “resource-matching effect”—the genetic characteristics of plants shape microbial communities that are favorable for their own ecological niche, and microbial communities in turn affect plant adaptation strategies [42]. Tariq et al. discovered that deep-rooted plants can not only quickly search for water sources through their main roots, but also transport deep water to shallow soil through “hydraulic lifting”, benefiting surrounding shallow-rooted plants and microorganisms. This has a “biological irrigation” effect, thereby reducing water competition within plant communities and promoting mutualistic symbiosis [43]. Moreover, fine-root traits are the primary factor shaping the rhizospheric *phoD*-harboring bacterial community, thereby affecting soil phosphorus availability. In contrast, the *phoD*-harboring bacterial community in non-rhizosphere soil is dominated by inorganic phosphorus. As plants grow, the importance of fine-root morphological characteristics for soil phosphorus availability gradually increases [44]. As Dini-Andreote et al. summarized, the microbiome could reshape root system architecture by synthesizing phytohormones (e.g., auxins, cytokinins, ethylene), clearing them from the plant’s immune system; this process is mediated by the bacterial alkaline protease AprA, which collaboratively absorbs nutrients by dissolving/transporting them (e.g., N, P) [45]. These findings highlight the synergistic variation patterns of leaf, stem, root traits, and plant microbiome and their response to climate change, herbivores, grazing, and so on. This helps us to better understand the driving factors of plant functional traits adapting to environmental changes at different latitudes in order to facilitate differentiated management of agricultural and forestry plants of different latitudes and types.
